# Changes in Practice Patterns of Clopidogrel in Combination with Proton Pump Inhibitors after an FDA Safety Communication

**DOI:** 10.1371/journal.pone.0145504

**Published:** 2016-01-04

**Authors:** Annie Guérin, Reema Mody, Valerie Carter, Charles Ayas, Haridarshan Patel, Karen Lasch, Eric Wu

**Affiliations:** 1 Analysis Group, Inc., Montreal, Quebec, Canada; 2 Takeda Pharmaceuticals International, Inc., Health Economics and Outcomes Research, Deerfield, Illinois, United States of America; 3 Immensity Consulting, Inc., Chicago, Illinois, United States of America; 4 Analysis Group, Inc., Boston, Massachusetts, United States of America; University of Milan, ITALY

## Abstract

**Objectives:**

In 2009, the FDA issued a warning that omeprazole–a proton pump inhibitor (PPI)–reduces the antithrombotic effect of clopidogrel by almost half when taken concomitantly. This study aims to analyze the impact of the FDA Safety Communications on prescribing clopidogrel together with PPIs.

**Methods:**

This retrospective study identified clopidogrel users from the Truven Health Analytics MarketScan Databases (01/2006–12/2012). Rates of clopidogrel-PPI combination therapy were estimated in 6-month intervals for patients with ≥1 clopidogrel prescription fill, then were analyzed pre- and post-safety communication (11/17/2009). Analyses were also conducted by grouping PPIs into CYP2C19 inhibitors (omeprazole and esomeprazole) and CYP2C19 non-inhibitors (pantoprazole, lansoprazole, dexlansoprazole, and rabeprazole).

**Results:**

Overall, 483,074 patients met the selection criteria; of these, 157,248 used a clopidogrel-PPI combination. On average, 30.5% of patients in the pre- and 19.9% in the post-communication period used a clopidogrel-PPI combination therapy. Among clopidogrel users, the probability of using clopidogrel-PPI combinations fell by over 40% in the post-communication period (OR = 0.57; *p*<0.001); the proportion of patients using esomeprazole fell from 12.9% to 5.3%, and the proportion using omeprazole fell from 10.1% to 6.3%. Among combination therapy users, the probability of patients using a combination with a CYP2C19 inhibitor decreased by 53% (OR = 0.47; *p*<0.001); however, 31.5% of patients were still prescribed a clopidogrel-PPI combination therapy. Trends were similar for all and newly treated patients, regardless of clopidogrel indication and physician specialty.

**Conclusions:**

The FDA Safety Communication resulted in a reduction in the number of patients undergoing combination therapy; however approximately one-third of patients still used combination therapy post-communication.

## Introduction

Clopidogrel, the second leading prescription drug sold worldwide in 2008, inhibits platelet aggregation by selectively and irreversibly binding to the adenosine diphosphate (ADP) receptors on platelets [1,2]. It is commonly prescribed as a prophylactic treatment, to prevent the formation and growth of thrombi in patients with recent myocardial infarctions, cerebrovascular accidents, acute coronary syndrome, or peripheral artery disease [1]. Clopidogrel has been associated with an increased risk of gastrointestinal (GI) bleeding [3]. Consequently, to reduce its associated risk of GI bleeding it is commonly prescribed in combination with other drugs that inhibit gastric acid production, such as proton pump inhibitors (PPIs) [4,5].

In 2006, a study by Gilard *et al*. raised concerns about a possible drug-drug interaction between clopidogrel and omeprazole (a PPI) that could result in a decreased efficacy of clopidogrel when taken in combination with omeprazole [6]. Clopidogrel is a pro-drug that is metabolically activated by the cytochrome p450 2C19 (CYP2C19); omeprazole (and its enantiomer, esomeprazole) is also metabolized through the same liver enzyme. Therefore, it is biologically plausible that the PPI may interfere with clopidogrel’s metabolism and attenuate its antiplatelet effects.

Subsequent trials and studies were conducted to test the hypothesis, but yielded mixed results. O’Donoghue *et al*. published in September 2009 a study concluding that their trial did not support the decision to avoid concomitant use of PPIs with clopidogrel [7]. Laine *et al*. reached a similar conclusion in a November 2009 publication [8]. In contrast to these studies, Gilard *et al*. published another study in 2008, concluding that, based on 124 patients enrolled in a double-blind placebo-controlled trial, omeprazole had a significant inhibitory effect on clopidogrel, as assessed by vasodilator-stimulated phosphoprotein phosphorylation test [9]. Other studies attempted to show whether the patients’ genetic backgrounds had an impact on the metabolism of clopidogrel and omeprazole, but these also yielded mixed conclusions [10,11].

This controversy prompted the Food and Drug Administration (FDA) to release an Early Communication about an ongoing safety review of clopidogrel in January 2009; at the time, product labels remained unchanged, due to insufficient evidence [6,9–12]. By November 2009, based on new findings from further studies performed by clopidogrel manufacturers, the FDA issued a Safety Communication stating that omeprazole reduced the antithrombotic effect of clopidogrel by almost half when taken concomitantly, and that concomitant use should be avoided [1,13]. Soon after the safety communication, clopidogrel manufacturers added package inserts emphasizing the drug interaction [1]. The FDA’s safety communication prompted further debate in the medical community, with the American College of Cardiology and the American Heart Association stating that some of the suggestions made by the FDA were unsupported by clinical evidence and that physicians should use clinical judgment in deciding the best approach for poor metabolizers of clopidogrel [14].

The clopidogrel-omeprazole interaction was also analyzed in a prospective clinical study, “Clopidogrel and Optimization of Gastrointestinal Events” (COGENT). The authors concluded that *“[t]here was no apparent cardiovascular interaction between clopidogrel and omeprazole*, *but [their] results do not rule out a clinically meaningful difference in cardiovascular events due to use of a PPI*” [15]. The FDA issued a reminder in October 2010 clarifying that the safety communication applied only to omeprazole, because not all PPIs have the same inhibitory effect on the enzyme CYP2C19 and, thus, other PPIs could be safely taken with clopidogrel [16].

To date, little is known about the impact of these FDA Safety Communications on prescription trends. The objective of this study is to analyze their impact on prescription trends for clopidogrel taken in combination with PPIs.

## Methods

### Data source

The analysis used data from the Truven Health Analytics MarketScan® Databases (Q1 2006 –Q4 2012), a private sector health data resource which reflects the healthcare experience of enrollees covered by the health benefit programs of large employers. Data are collected from over 100 different insurance companies. The data represent the medical claims of insured employees and their dependents, as well as Medicare-eligible retirees with employer-provided Medicare supplement plans. For this type of study formal consent is not required (study is exempt from Institutional Review Board approval) since patient information has been anonymized and de-identified prior to being delivered to the investigators. The Truven Health Analytics MarketScan® Databases are fully compliant with the Health Insurance Portability and Accountability Act (HIPAA) of privacy.

### Study design

The study period was January 2006—December 2012 (see S1 Fig for timeline of FDA safety communications and PPI label updates), with each calendar year divided into 6-month intervals (semesters). The pre- and post-FDA Safety Communication periods were defined as 2006 (S1)– 2009 (S1) and 2009 (S2)– 2012 (S2), respectively. The post-FDA Safety Communication period was further divided into the post-FDA reminder period 2011 (S1)– 2011 (S2) and the post-label change period 2012 (S1)– 2012 (S2). The rationale for this subdivision was concern over the impact of the label changes not being felt directly (either after the safety communication or after the FDA reminder), and that the results might be subject to transitory noise.

Each semester was analyzed cross-sectionally, based on patients meeting the following selection criteria during that study semester: they were at least 18 years of age as of the beginning of the given semester, had continuous health plan enrollment during the given and previous semester as well as through 2009 (S1)– 2010 (S1), and had at least one prescription fill for clopidogrel during the given semester. All patients meeting the above selection criteria were defined as clopidogrel users for the given semester.

Among clopidogrel users, combination therapy users were defined as patients who had at least one prescription fill for a PPI with an overlap of at least 30 days with the clopidogrel prescription during that semester. All aspects of study design, data analysis, and publication follow STROBE guidelines for observational study reporting, as detailed in S2 Table.

### Measures and outcomes

Patient characteristics, including age, gender, potential indications for the use of clopidogrel, prescribing physician’s specialty, and Charlson comorbidity index [17] (CCI; higher index scores indicate greater comorbidity burden) were reported as of the initiation of therapy for both the clopidogrel users and the combination therapy users.

Rates of combination therapies were calculated for each semester between 2006 (S1) and 2012 (S2). Rates of combination therapies for a given semester were calculated using the number of combination therapy users during the studied semester as the numerator and the number of clopidogrel users during the same semester as the denominator. Rates of combination therapies were also calculated separately for each PPI. The rates of combination therapies by PPI among clopidogrel users were calculated using the number of patients using clopidogrel during the studied semester in combination with the studied PPI as the numerator, and the number of clopidogrel users during the same semester as the denominator. Finally, relative proportions of combination users by each PPI among all combination therapy users were calculated using the number of patients using clopidogrel during the studied semester in combination with the studied PPI as the numerator and the number of combination therapy users during the same semester as the denominator.

For the analyses of the relative proportions of combination users by each PPI among all combination therapy users and the rates of combination therapies by PPI among all clopidogrel users, PPIs were analyzed individually and combined by whether the PPI has a large inhibitor effect or not. CYP2C19 inhibitors included omeprazole and esomeprazole, and CYP2C19 non-inhibitors included dexlansoprazole, lansoprazole, pantoprazole, and rabeprazole [18,19].

### Statistical analyses

Patient characteristics were descriptively reported as of the initiation of therapy for both clopidogrel users and combination therapy users. The proportion of patients using each clopidogrel-PPI combination therapy among all clopidogrel users and among all clopidogrel-PPI combination therapy users was also descriptively reported.

Logistic regression models were used to test whether the likelihood of being prescribed a combination therapy changed before and after the 2009 FDA Safety Communication. This analysis was replicated for the pre-safety communication period compared to the post-label change period and for the post-FDA reminder period compared to the post-label change period. For the relative proportions of combination users by each PPI among all combination therapy users and the rates of combination therapies by PPI among clopidogrel users, the regressions were performed strictly on the whole sample.

### Subgroup analyses

Subgroup analyses were conducted by potential indications for the use of clopidogrel. Because the diagnosis was not recorded with prescription claims, proxies of potential indications were identified based on diagnoses recorded prior to therapy initiation. Potential indications included acute coronary syndrome, recent myocardial infarctions (diagnosis within 35 days of therapy), peripheral artery disease, recent strokes (diagnosis within 6 months of therapy) and other ischemic heart diseases.

Subgroup analyses were also conducted by prescribing physician specialty (cardiologists and gastroenterologists; all other specialties were grouped in an ‘other’ category, whose results were not shown). Similarly, since the prescriber specialty was not available in the data source, the specialty of the prescribing physician was defined based on the specialty recorded in the last medical encounter within the 30 days before the therapy initiation. Another subgroup consisted of new users, defined as patients who did not use any anti-platelet ADP receptor inhibitor medication during the 6-month period preceding the studied interval.

## Results

From 2006 (S1) to 2012 (S2), 483,074 unique clopidogrel users and 157,248 unique combination therapy users met the inclusion criteria for at least one semester, of which 39.0% and 57.0% were women. Average ages for the groups were 65.3 and 67.4 years, respectively (**Table 1**).

**Table 1:** Characteristics of patients initiated on clopidogrel therapy and clopidogrel-PPI combination therapy.

Baseline characteristics	Clopidogrel therapy (N = 483,074)	Combination therapy (N = 157,248)
***Demographic characteristics ***		
Age, mean ± SD [median]	65.3 ± 12.3 [64.0]	67.4 ± 11.8 [67.0]
Female, N (%)	188,362 (39.0%)	89,666 (57.0%)
***Potential indication for the use of anti-platelet ADP receptor inhibitor*, *N (%) ***		
Acute coronary syndrome	118,275 (24.5%)	41,747 (26.5%)
Peripheral arterial disease	169,293 (35.0%)	60,257 (38.3%)
Recent myocardial infarction	91,969 (19.0%)	23,369 (14.9%)
Recent stroke	185,897 (38.5%)	63,957 (40.7%)
Other ischemic heart disease indication	368,363 (76.3%)	125,622 (79.9%)
***Prescribing physician specialty*, *N (%) ***		
Cardiology	196,713 (40.7%)	55,100 (35.0%)
Gastroenterology	19,705 (4.1%)	12,078 (7.7%)
***Charlson-Quan comorbidity index*, *mean ± SD [median]***	2.7 ± 2.4 [2.0]	3.4 ± 2.6 [3.0]

Abbreviations: ADP, adenosine diphosphate; PPI, proton pump inhibitor; SD, standard deviation.

The average CCI was 2.7 and 3.4 among the clopidogrel users and the combination therapy groups, respectively (**Table 1**). Over three-quarters of patients in both groups had indications for other ischemic heart diseases (76.3% and 79.9%, respectively), and 38.5% and 40.7%, respectively, suffered recent strokes (**Table 1**).

**Fig 1:**
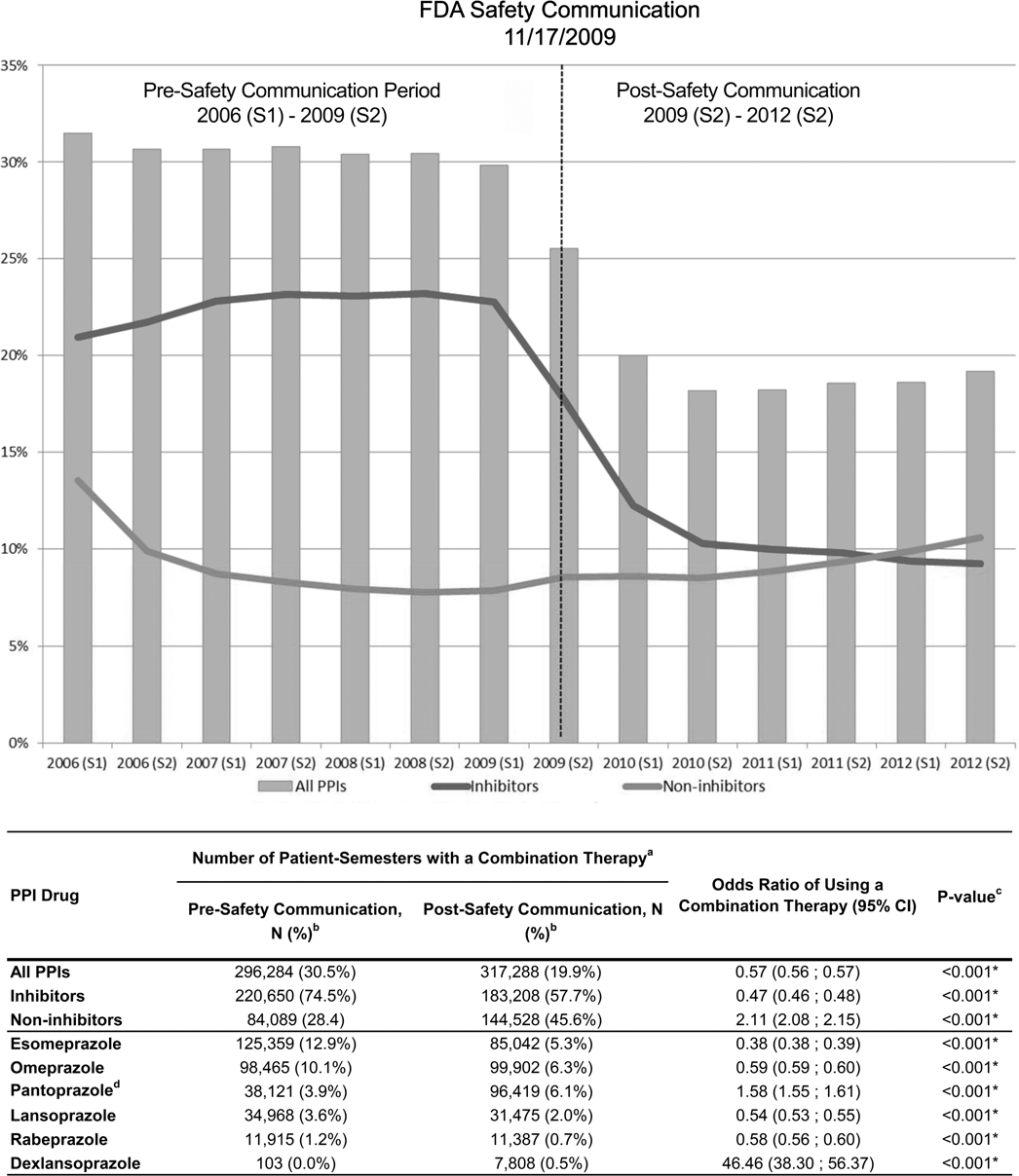
Among clopidogrel users, proportion of patients using clopidogrel-PPI combination therapy over time. Notes: ^a^ Total number of patient-semesters pre-safety communication = 272,113; post-safety communication = 1,593,625. ^b^ Percentages add up to more than 100% because more than 1 PPI could be prescribed in the same semester. ^c^ * indicates that the results are significant at the 5% level (two-sided alpha). ^d^ Protonix was first released as a generic, pantoprazole, in August 2007. Abbreviations: CI, confidence interval; PPI, proton pump inhibitor; S, semester.

Since each semester was analyzed cross-sectionally, patients were not observed over time. Instead, patient intervals were analyzed, and patients may appear in more than one semester of the study period. A total of 972,113 pre- and 1,593,625 post-safety communication patient intervals were identified. For brevity, intervals will be omitted in future reporting. On average, 30.5% of patients in the pre-safety-communication period and 19.9% in the post-safety-communication period used a clopidogrel-PPI combination therapy. The probability of using a clopidogrel-PPI combination therapy fell more than 40% post safety communication (OR = 0.57; *p* < 0.001). However, starting from 2011, there seemed to be an increasing trend in the proportion of patients using a clopidogrel-PPI combination therapy (**Fig 1**).

When comparing the likelihood of using a clopidogrel-PPI combination therapy in 2012 (S2) vs. 2010 (S2), there was a 7% higher likelihood of use in 2012 (OR = 1.07; *p* < 0.001 results not shown).

Among all clopidogrel users, the percentage of patients using omeprazole and esomeprazole dropped substantially during the post-safety-communication period compared to the pre-safety-communication period (12.9% vs. 5.3% and 10.1% vs. 6.3% respectively). Overall, the proportion of patients using clopidogrel in combination with a CYP2C19 inhibitor PPI dropped 66% (OR = 0.44; *p* < 0.001). On the other hand, the percentage of patients using a combination with a CYP2C19 non-inhibitor PPI increased 5% during the post-safety communication period (OR = 1.05; *p* < 0.001). Pantoprazole and dexlansoprazole were the only PPIs with a significant increased use post-safety communication (3.9% vs. 6.1% and 0.0% vs. 0.5%, respectively) (**Fig 1**).

Among all combination therapy users, the probability of patients using omeprazole only decreased 8% compared to the pre-safety-communication period (33.2% vs. 31.5% for the pre- vs. the post-communication period; OR = 0.92; *p* < 0.001), while the probability of patients using esomeprazole decreased by half (42.3% vs. 26.8% for the pre- vs. the post-communication period; OR = 0.502; *p* < 0.001) (**Table 2**).

**Table 2:** Among combination therapy users, relative proportions of each PPI: pre- vs. post- safety communication^a^ - (≤ 2009 S1 vs. ≥ 2009 S2).

	Number of Patient-Semesters^a^ with a Combination Therapy		Odds Ratio of Using a Combination	P-value^c^
PPI Drug	Pre-Safety Communication, N (%)^b^	Post-Safety Communication, N (%)^b^	Therapy (95% CI)	
**Inhibitors**	220,650 (74.5%)	183,208 (57.7%)	0.47 (0.46; 0.48)	<0.001*
**Non-inhibitors**	84,089 (28.4%)	144,528 (45.6%)	2.11 (2.08; 2.15)	<0.001*
**Esomeprazole**	125,359 (42.3%)	85,042 (26.8%)	0.50 (0.49; 0.51)	<0.001*
**Omeprazole**	98,465 (33.2%)	99,902 (31.5%)	0.92 (0.91; 0.94)	<0.001*
**Pantoprazole**^**d**^	38,121 (12.9%)	96,419 (30.4%)	2.96 (2.90; 3.02)	<0.001*
**Lansoprazole**	34,968 (11.8%)	31,475 (9.9%)	0.82 (0.80; 0.84)	<0.001*
**Rabeprazole**	11,915 (4.0%)	11,387 (3.6%)	0.89 (0.85; 0.92)	<0.001*
**Dexlansoprazole**	103 (0.0%)	7,808 (2.5%)	72.55 (59.80; 88.02)	<0.001*

**Notes**:

^a^ Total number of patient-semesters pre-safety communication = 296,284; post-safety communication = 317,288.

^b^ Percentages add up to more than 100% because more than 1 PPI could be prescribed in the same semester.

^c^ * indicates that the results are significant at the 5% level (two-sided alpha).

^d^ Protonix was first released as a generic, pantoprazole, in August 2007.

Abbreviations: CI, confidence interval; PPI, proton pump inhibitor; S, semester.

Patients using a clopidogrel-PPI combination therapy were 53% less likely to use a combination with a CYP2C19 inhibitor during the post-safety-communication period compared to before. Patients transferring from CYP2C19 inhibitors mainly took the non-inhibitor PPIs pantoprazole and dexlansoprazole. The proportion of patients using a combination with pantoprazole increased almost three-fold in the post- vs. the pre-safety-communication period (OR = 2.96; *p* < 0.001) (**Table 2**). Dexlansoprazole also had a significant increase in the frequency of prescription (0.0% vs. 2.5%), the probability of which increased dramatically (OR = 72.55; *p* < 0.001) (**Table 2**). However, it should be noted that dexlansoprazole was initially approved in January 2009, making it difficult to establish a link between the increased use and the FDA Safety Communication.

The decrease in the probability of using a clopidogrel-PPI combination post- vs. pre-safety-communication was observed in each subgroup. When stratifying the analysis by potential indication for clopidogrel, the decrease in probability ranged from 37% for patients who suffered recent myocardial infarctions to 48% for patients who suffered acute coronary syndrome (**Table 3**).

**Table 3:** Subgroup analysis of patients initiated on clopidogrel therapy (A) and clopidogrel combination therapy (B)—(≤ 2009 S1 vs. ≥ 2009 S2).

Potential indication for the use of clopidogrel	Number of Patient-Semesters with a Combination Therapy				Odds Ratio of Using a Combination Therapy (95% CI)	P-value^a^
	(A) Pre-Safety Communication, N	(A) Post-Safety Communication, N	(B) Pre-Safety Communication, N (%)	(B) Post-Safety Communication, N (%)		
**Acute coronary syndrome**	103,928	142,899	35,750 (34.4%)	30,725 (21.5%)	0.52 (0.51; 0.53)	<0.001*
**Peripheral arterial disease**	168,267	322,827	55,611 (33.0%)	66,924 (20.7%)	0.53 (0.52; 0.54)	<0.001*
**Recent myocardial infarction**	46,291	75,196	10,993 (23.7%)	12,307 (16.4%)	0.63 (0.61; 0.65)	<0.001*
**Recent stroke**	148,146	262,174	46,418 (31.3%)	52,476 (20.0%)	0.55 (0.54; 0.56)	<0.001*
**Other ischemic disease**	610,731	1,043,106	192,822 (31.6%)	213,694 (20.5%)	0.56 (0.55; 0.56)	<0.001*
**Cardiology**	127,276	178,392	32,256 (25.3%)	31,946 (17.9%)	0.64 (0.63; 0.66)	<0.001*
**Gastroenterology**	8,237	14,030	4,740 (57.5%)	6,743 (48.1%)	0.68 (0.65; 0.72)	<0.001*
**New users**	161,845	211,914	34,034 (21.0%)	31,271 (14.8%)	0.65 (0.64; 0.66)	<0.001*

Notes

^a^ * indicates that the results are significant at the 5% level (two-sided alpha).

Abbreviations: CI, confidence interval; PPI, proton pump inhibitor; S, semester.

Patients were generally seen by a cardiologist– 40.7% for clopidogrel users and 35.0% for combination therapy users–and only 4.1% and 7.7%, respectively, were seen by a gastroenterologist (**Table 1**). Regardless of physician specialty, there was a decline in the proportion of patients using combination therapy post-safety communication (gastroenterologist: OR = 0.64; and cardiologist: OR = 0.68; all *p* < 0.001) (**Table 3**). A similar trend was observed in new users of clopidogrel (OR = 0.65; *p* < 0.001) (**Table 3**).

## Discussion

In November 2009, the FDA issued a safety communication calling for the avoidance of concomitant use of clopidogrel and omeprazole or esomeprazole, due to potential for drug-drug interactions [12]. This study found that, following the issuance of the safety communication, the proportion of patients receiving clopidogrel-PPI combination therapy substantially decreased. However, the proportion of patients using clopidogrel in combination with omeprazole remained high in existing combination therapy users.

Despite the mention of only omeprazole and esomeprazole in the safety communication, arguments arose in the medical community questioning the validity of the FDA warning [14] and the use of any PPIs in combination with clopidogrel [20]. As a consequence, in October 2010, the FDA issued a reminder clarifying that the safety communication applied only to omeprazole (no mention of esomeprazole), since PPIs that are weaker inhibitors of CYP2C19 do not interact with clopidogrel to the same degree and, thus, could be safely taken with clopidogrel [13,16]. Following these communications, a shift from omeprazole to other PPIs that do not inhibit the CYP2C19 system might have been expected. In the current study, the main decreases were observed for esomeprazole, followed by lansoprazole; which were partially replaced by pantoprazole and dexlansoprazole.

In this study, we observed that omeprazole prescriptions declined and then plateaued, whereas esomeprazole prescriptions steadily declined (results not presented). These results suggest that the change in prescription trends may reflect two distinct effects: the FDA communication effect and the generic effect. First, the FDA Safety Communication may have led to a decrease in the proportion of patients who were prescribed a CYP2C19 inhibitor in combination with clopidogrel. After several months, we observed that the proportion of patients being prescribed omeprazole in combination with clopidogrel stabilized, while the proportion of patients being prescribed esomeprazole in combination with clopidogrel continued to decline. This may be partially attributed to a generic substitution effect; while omeprazole had been available as a generic since 2002, esomeprazole was just released as a generic in 2014 (data for this study were only available until end of 2012). When the FDA released a reminder in October 2010, suggesting pantoprazole as a viable alternative [16], physicians may have been more inclined to switch patients or to start prescribing clopidogrel combination therapy with a treatment that was available as a generic (the generic version of pantoprazole had been available since 2008, see S1 Table).

Pantoprazole and dexlansoprazole are the only two PPIs that had an increase in their prescription trends during the post-safety communication period. Their increased use may be explained by clinical practice updates based on FDA communications, clinical studies, and product launches. Indeed, in October 2010, the FDA released a reminder warning against only omeprazole in combination with clopidogrel and suggesting pantoprazole as an alternative [16]. In October 2011 the pantoprazole and dexlansoprazole drug labels were changed to indicate no important clinical impact on clopidogrel metabolism [21,22]. In addition, studies published in 2010 [23] and 2011 [24] for pantoprazole and in 2012 for dexlansoprazole [25] showed reduced metabolic drug-drug interaction with clopidogrel, compared to omeprazole. Finally, dexlansoprazole was launched in 2009, and market share progressively increased.

Among all clopidogrel users, although there has been a decrease in the proportion of patients receiving a combination with a CYP2C19 inhibitor PPI, for those who continued to take combination therapies, omeprazole and esomeprazole remained the second and third most prescribed PPIs, behind pantoprazole, until the end of 2011. Continued high use of omeprazole and esomeprazole could be explained by the mixed results that were published in the literature and the lack of strong belief in the risk of this drug-drug interaction in community practice. When the safety communication was released, one study questioned the validity of the FDA warning, noting that it was based largely on ex vivo data and not clinical trials [26]. In addition, a prospective clinical study showed that the clopidogrel-omeprazole combination resulted in a significant decrease in GI events, but not in a significant increased risk of cardiovascular events [15,27]. However, the FDA disregarded these new findings from the trial, given its limitations [28].

Finally, the debate on the possible negative impact of administration of PPIs in conjunction with antiplatelet medication on cardiovascular function is still ongoing. Two separate research groups have recently examined findings from prior randomized trials and observational studies in a systematic literature review [29] and a meta-analysis [30] and have concluded that, while some observational studies may show an increased cardiovascular risk in patients co-administered antiplatelet medication and a PPI, randomized controlled trials show no such link. Cardoso et al. (2015) have further suggested that the observation of increased cardiovascular risk in PPI-clopidogrel-treated patients in observational studies reflects the use of clopidogrel in a population with higher cardiovascular comorbidity at baseline, rather than a true pharmacodynamic interaction between PPIs and clopidogrel [30].These recent findings warrant further monitoring of PPI-clopidogrel prescription trends in the years to come.

This study is subject to some limitations. First, it is difficult to distinguish the effect of the FDA Safety Communication from other effects, such as the changes in the regulatory environment, new entrants into the market, patent expiry and the entry of generics, and possibly other market changes. Around the time of the safety communication, two new PPIs entered the market and the generic version of pantoprazole became available. In addition, prasugrel (another anti-platelet agent) was available in the same period as a clopidogrel alternative, and since it does not interact with the CYP2C19 system, it may have been prescribed instead of clopidogrel [31]. Second, this analysis was based on longitudinal data that were analyzed strictly cross-sectionally; that is, each period was observed individually and independently of the previous periods. Hence, there is no information on whether patients continued treatments, switched to different therapies, or discontinued prior therapies. Third, it is impossible to determine if patients stopped combination therapy in order to switch to a non-prescription OTC medication or to an H2 blocker. Fourth, claims databases do not include the rationale behind therapeutic decisions; therefore it is difficult to determine the true intent of prescribers. Finally, this study was subject to the common limitations of retrospective, observational studies based on healthcare claims data, such as errors and omissions in the database, as well as lack of ability to capture the over-the-counter use of PPIs. Nevertheless, these limitations would affect all patients to a similar extent, and are thus unlikely to have altered the results.

Historically, FDA label change recommendations have been shown to impact the prescription market. Clinical studies on antidepressants, antipsychotics, antiemetics, and immunoglobulin therapies have all similarly shown trends toward decreases in prescribing after the FDA released a warning that resulted in a label change [32–35]. Even in cases of clinical interpretation controversy, a similar effect was seen in a study on the antiemetic and antipsychotic droperidol. Habib *et al*. suggested that even though surveyed practitioners did not believe the FDA Safety Communication regarding droperidol was justified, the use of the agent still declined [33]. Since the potential adverse effects of some drug interactions may increase the burden to payers and prove to be harmful to the patient’s health [36–38], a clinician or a payer may find it more expedient to switch to an alternative therapy even before full judgment can be rendered.

To conclude, this analysis highlights two important facts: 1) the FDA Safety Communication resulted in a reduction in the total number of patients undergoing clopidogrel-PPI combination therapy and 2) although a decrease in the proportion of patients receiving a combination with a CYP2C19 inhibitor PPI, omeprazole remained one of the PPIs most prescribed with clopidogrel. These findings are surprising considering the multitude of FDA communications, drug label changes, and clinical studies discouraging the use of such combination.

## Supporting Information

S1 Fig Chronology of Events during Study Period. Abbreviations: FDA, United States Food and Drug Administration; PPI, proton pump inhibitors.
(DOCX)

S1 Table Chronology of generic entries into the market. * Note that Teva Pharmaceuticals launched a generic pantoprazole in 2007 but legal action was taken by Wyeth Pharmaceuticals for patent infringement. Wyeth's marketing exclusivity expired January 2011 but in response to Teva, Wyeth released a generic pantoprazole in January 2008.Abbreviations: OTC, over the counter.
(DOCX)

S2 Table STROBE Statement—checklist of items that should be included in reports of observational studies. (DOC)
