# Supplementary material for: Changes in Practice Patterns of Clopidogrel in Combination with Proton Pump Inhibitors after an FDA Safety Communication
Source: PLoS One. 2016 Jan 4;11(1):e0145504. doi: 10.1371/journal.pone.0145504 (PMC4699636; doi:10.1371/journal.pone.0145504)
Supplement: S1 Fig — Abbreviations: FDA, United States Food and Drug Administration; PPI, proton pump inhibitors. (DOCX) [file pone.0145504.s001.docx]

## S1 Figure: Chronology of Events during Study Period

Abbreviations: FDA, United States Food and Drug Administration; PPI, proton pump inhibitors.
