# Supplementary material for: Changes in Practice Patterns of Clopidogrel in Combination with Proton Pump Inhibitors after an FDA Safety Communication
Source: PLoS One. 2016 Jan 4;11(1):e0145504. doi: 10.1371/journal.pone.0145504 (PMC4699636; doi:10.1371/journal.pone.0145504)
Supplement: S1 Table — * Note that Teva Pharmaceuticals launched a generic pantoprazole in 2007 but legal action was taken by Wyeth Pharmaceuticals for patent infringement. Wyeth's marketing exclusivity expired January 2011 but in response to Teva, Wyeth released a generic pantoprazole in January 2008.Abbreviations: OTC, over the counter. (DOCX) [file pone.0145504.s002.docx]

## S1 Table: Chronology of generic entries into the market

| **Chronology of generic entries into the market** | | | |
| --- | --- | --- | --- |
| **Brand** | **Generic** | **Generic release date** | **OTC** |
| Plavix | Clopidogrel | May 2012 | No |
| Prilosec | Omeprazole | November 2002 | Yes -2003 |
| Nexium | Esomeprazole | - | Yes - 2014 |
| Dexilant | Dexlansoprazole | - | No |
| Prevacid | Lansoprazole | November 2009 | Yes - 2009 |
| Protonix | Pantoprazole | January 2008* | No |
| Aciphex | Rabeprazole | November 2013 | No |

* Note that Teva Pharmaceuticals launched a generic pantoprazole in 2007 but legal action was taken by Wyeth Pharmaceuticals for patent infringement. Wyeth's marketing exclusivity expired January 2011 but in response to Teva, Wyeth released a generic pantoprazole in January 2008.

Abbreviations: OTC, over the counter.
